# Prevalence and Determinants of Malaria Among Children Under Five Years in Malaria‐Endemic Areas of Mpumalanga Province, South Africa

**DOI:** 10.1155/ghe3/7878182

**Published:** 2026-07-29

**Authors:** Masilu Daniel Masekameni, Themba Titus Sigudu, Khathutshelo Vincent Mphaga, Phoka Caiphus Rathebe

**Affiliations:** ^1^ Development Studies, University of South Africa, Pretoria, South Africa, unisa.ac.za; ^2^ Division of Health and Society, University of the Witwatersrand, Johannesburg, South Africa, wits.ac.za; ^3^ Department of Environmental Health, University of Johannesburg, Johannesburg, South Africa, uj.ac.za

**Keywords:** children under five, household characteristics, malaria, malaria prevention, Mpumalanga province, sociodemographic determinants, socioeconomic factors

## Abstract

**Background:**

Malaria remains a major public health concern among children under five in sub‐Saharan Africa, with sociodemographic and environmental factors influencing transmission. This study assessed malaria prevalence and examined sociodemographic, socioeconomic, household and malaria prevention determinants associated with malaria risk among children under five in malaria‐endemic areas of Mpumalanga Province.

**Methods:**

A cross‐sectional analytical study was conducted using secondary data obtained from the Demographic and Health Surveys (DHS) Programme collected from malaria‐endemic areas of Mpumalanga Province, South Africa. The study included 108,898 children aged 0–59 months with complete information on malaria status and relevant explanatory variables. Data were collected using standardised household questionnaires and sampling procedures employed by the DHS Programme. Descriptive statistics and logistic regression analyses were performed to identify sociodemographic, socioeconomic, household and malaria prevention factors associated with malaria infection. Crude odds ratios (CORs) and adjusted odds ratios (AORs) with 95% confidence intervals (CIs) and *p* values of ≤ 0.05 indicate a significant statistical difference, while a > 0.05 *p* value reporting no statistical significance.

**Results:**

The overall malaria prevalence was 4.91% (95% CI: 4.78–5.04). Higher prevalence was observed in rural areas (6.40%) compared to urban areas (1.44%). Children from the poorest households (8.00%) and those with mothers with no formal education (9.40%) had higher malaria prevalence. Nkomazi had the highest burden (12.25%). In multivariable analysis, rural residence (AOR = 2.35; 95% CI: 2.10–2.63; *p* < 0.001), no maternal education (AOR = 2.05; 95% CI: 1.75–2.40; *p* < 0.001), poorest wealth status (AOR = 2.48; 95% CI: 2.10–2.93; *p* < 0.001), nonuse of insecticide‐treated nets (AOR = 1.72; 95% CI: 1.55–1.92; *p* < 0.001) and absence of indoor residual spraying exposure (AOR = 1.31; 95% CI: 1.17–1.47; *p* < 0.001) were significant predictors of malaria infection.

**Conclusion:**

Malaria risk is strongly influenced by socioeconomic disadvantage, rural residence and inadequate prevention measures. Targeted interventions addressing inequality and improving coverage and utilisation of malaria control strategies are essential.

## 1. Introduction

Malaria remains a major global public health challenge, disproportionately affecting populations in sub‐Saharan Africa despite decades of intensified control efforts. The most recent estimates indicate approximately 249 million malaria cases and 608,000 deaths globally, with over 94% of cases occurring in the African region [1]. Children under five years of age bear the greatest burden, accounting for nearly 80% of malaria‐related mortality, largely due to their immature immunity and increased vulnerability to severe disease [1, 2]. Sub‐Saharan Africa continues to carry the greatest burden of malaria globally, with children under five years accounting for the majority of malaria‐related morbidity and mortality, while South Africa continues to experience persistent transmission in endemic provinces such as Mpumalanga, Limpopo and KwaZulu‐Natal despite ongoing malaria elimination efforts [1, 2]. Although substantial gains were achieved between 2000 and 2015, progress has plateaued in recent years, with emerging challenges including insecticide resistance, climate variability and persistent socioeconomic inequalities undermining control efforts [1, 3]. Malaria transmission is shaped by a complex interplay of environmental, biological and sociodemographic factors. Increasingly, evidence highlights the critical role of social determinants of health, including poverty, education and access to preventive interventions, in influencing malaria risk and outcomes [4–6]. Children from low‐income households are more likely to live in environments conducive to mosquito breeding, have limited access to healthcare services and experience barriers to effective prevention and treatment [5, 7]. Similarly, maternal education has been consistently associated with improved malaria prevention practices, timely care‐seeking and reduced child morbidity [6, 8]. Rural residence further compounds these risks, as rural communities often face reduced access to health infrastructure, lower intervention coverage and higher vector exposure [9].

Malaria prevention strategies, particularly insecticide‐treated nets (ITNs) and indoor residual spraying (IRS), remain central to global malaria control programmes. While significant scale‐up of these interventions has occurred, gaps persist in both coverage and utilisation. Evidence suggests that ownership of ITNs does not always translate into consistent use, and IRS programmes often fail to reach all at‐risk households, thereby limiting their effectiveness [10–12]. These gaps are particularly pronounced in socioeconomically disadvantaged and rural settings, where the malaria burden remains highest [11]. In South Africa, malaria transmission is largely confined to the north‐eastern provinces of Mpumalanga, Limpopo and KwaZulu‐Natal, with seasonal and focal transmission patterns [13]. The country has made notable progress towards malaria elimination; however, persistent transmission in border regions continues to pose a significant challenge. The Ehlanzeni District in Mpumalanga Province, particularly the Nkomazi subdistrict, remains a hotspot due to its proximity to Mozambique, facilitating cross‐border importation of malaria cases [14, 15]. This spatial heterogeneity highlights the need for targeted, context‐specific interventions that address both local transmission dynamics and broader socioeconomic determinants.

Despite growing recognition of the importance of sociodemographic factors, there remains a paucity of subnational evidence examining how these determinants interact to influence malaria risk among vulnerable populations in South Africa. Most studies have focused on national‐level analyses or broader regional trends, with limited attention to high‐burden districts and localised risk patterns [6, 16]. Furthermore, changes in socioeconomic conditions, population movement and intervention coverage necessitate updated analyses using recent, high‐quality datasets. The Demographic and Health Surveys (DHS) provide a valuable source of nationally representative data, enabling robust assessment of health outcomes and their determinants across diverse settings [17]. Leveraging such data allows for a comprehensive evaluation of both household‐level and individual‐level factors associated with malaria risk [18].

Therefore, this study assessed malaria prevalence and examined sociodemographic, socioeconomic, household and malaria prevention determinants associated with malaria risk among children under five in malaria‐endemic areas of Mpumalanga Province. By identifying key risk factors, this study seeks to inform targeted interventions, support evidence‐based policy and contribute to ongoing malaria elimination efforts in South Africa and similar settings.

## 2. Materials and Methods

### 2.1. Study Design and Data Source

This study employed a cross‐sectional analytical design using secondary household‐level data obtained from the DHS Programme. The DHS provides nationally representative data on population health, sociodemographic characteristics and malaria‐related indicators, collected using standardised questionnaires and rigorous sampling procedures. The DHS Programme employs a stratified two‐stage cluster sampling design, in which enumeration areas (EAs) are selected during the first stage, followed by systematic selection of households within each cluster during the second stage.

### 2.2. Sampling Technique

In the initial stage, the sampling frame was stratified according to administrative regions and residential settings (urban and rural), subsequent to which EAs were selected through probability proportional to size sampling techniques. In the subsequent stage, a comprehensive household listing was executed within each chosen EA, and households were systematically chosen utilising equal probability sampling methods. All eligible households and respondents within the chosen households were incorporated in accordance with the DHS survey protocol. The application of this sampling methodology guaranteed that the study sample accurately reflected the target population and facilitated the reliable estimation of population‐level indicators.

### 2.3. Study Setting

The study was conducted in malaria‐endemic areas of Mpumalanga Province, located in the north‐eastern part of South Africa. Mpumalanga shares international borders with Mozambique and Eswatini, making it a key region for malaria transmission and control within the country. Malaria transmission in the province is typically seasonal, with peak incidence occurring during the rainy months (October–May), when environmental conditions favour mosquito breeding and parasite development. The analysis focused on the Ehlanzeni District, which is the primary malaria‐endemic district in Mpumalanga. This district comprises several localities, including Nkomazi, Mbombela, Umjindi (Barberton) and areas within the Kruger National Park (KNP) and surrounding lodges. Among these, Nkomazi is recognised as the highest transmission area, largely due to its proximity to the Mozambique border, which facilitates cross‐border movement of populations and importation of malaria cases.

The district is predominantly rural, with communities characterised by varying levels of socioeconomic development, housing conditions and access to basic services.

### 2.4. Study Population and Sample Size

The study population comprised children under five years of age (0–59 months) residing in malaria‐endemic areas of Mpumalanga Province. A total of 108,898 children were included in the analysis, representing the population at risk of malaria in these endemic settings. The analysis included all children with complete information on malaria status and relevant explanatory variables, including sociodemographic, socioeconomic, household and malaria prevention indicators. Records with missing or incomplete data on key variables were excluded to ensure the robustness and validity of the analysis. This approach enabled a comprehensive assessment of malaria prevalence and determinants among children living in areas where transmission persists.

### 2.5. Variables and Measurements

#### 2.5.1. Outcome Variable

The primary outcome variable was malaria infection status among children under five years of age, defined based on reported malaria cases in the DHS dataset. Malaria prevalence was calculated as the proportion of children with malaria relative to the total study population.

#### 2.5.2. Independent Variables

Independent variables were grouped into three categories. Demographic characteristics included child age group, sex, place of residence (rural or urban) and locality. Socioeconomic characteristics comprised maternal education level, household wealth index, access to electricity, source of drinking water and type of sanitation facility. Malaria prevention and household factors included ownership of ITNs, whether the child slept under an ITN the night preceding the survey, and exposure to IRS within the past 12 months. Improved water sources included piped water, boreholes, protected wells and protected springs, while unimproved sources included unprotected wells, surface water and other unsafe sources. Improved sanitation facilities included flush toilets and ventilated improved pit latrines, whereas unimproved sanitation referred to facilities that did not hygienically separate human waste from human contact.

### 2.6. Data Analysis

Data were analysed using Stata Statistical Software: version 20 (College Station, TX: StataCorp LLC; 2025). Descriptive statistics were used to summarise demographic, socioeconomic, household and malaria prevention characteristics of the study population. Categorical variables were presented as frequencies and percentages with corresponding 95% confidence intervals (CIs). Malaria prevalence was estimated overall and stratified by key explanatory variables, including place of residence, maternal education level, household wealth index and locality.

Associations between independent variables and malaria infection were first assessed using bivariate logistic regression analysis, with results presented as crude odds ratios (CORs), 95% CIs and *p* values. Variables with statistical significance at *p* < 0.05, as well as those identified a priori from the literature as potential determinants, were considered for inclusion in the multivariable model. A multivariable logistic regression model was subsequently fitted to identify independent predictors of malaria infection. Results were reported as adjusted odds ratios (AORs) with 95% CIs and *p* values, controlling for potential confounders including age group, sex, place of residence, maternal education, household wealth index, locality, ITN use and IRS exposure.

Multicollinearity among independent variables was assessed using variance inflation factors (VIFs), with no evidence of significant multicollinearity observed. Model goodness‐of‐fit was evaluated using the Hosmer–Lemeshow goodness‐of‐fit test to assess the adequacy of the final multivariable logistic regression model. All statistical tests were two‐sided, and statistical significance was set at *p* < 0.05.

### 2.7. Ethical Considerations

Ethical approval for this study was granted by the University of South Africa (UNISA), College of Agriculture and Environmental Sciences Human Research Ethics Committee (CAES‐HREC) (Ref: 2025/CAES_HREC/11764). Authorisation to access and analyse the DHS datasets was obtained from the DHS Programme, administered by ICF International, following registration of the research project within the DHS data access system. The DHS datasets are anonymised public‐use data, with no personal identifiers such as names or household addresses. Geographical information is restricted to broad administrative levels, and EA coordinates are displaced to protect confidentiality. All analyses adhered to the DHS data‐use agreement and relevant South African legislation, including the Protection of Personal Information Act (POPIA), with results presented in aggregated form to ensure privacy.

## 3. Results

### 3.1. Demographic Characteristics of Children Under Five

A total of 108,898 children under five years of age were included in the analysis (Table 1). The age distribution was relatively even across categories, with a slight increase in the proportion of older children. Children aged 48–59 months constituted the largest group (22.7%; 95% CI: 22.5–22.9), followed by those aged 36–47 months (21.6%; 95% CI: 21.4–21.8) and 24–35 months (20.7%; 95% CI: 20.5–20.9). The youngest age group, 0–11 months, accounted for 16.0% (95% CI: 15.8–16.2) of the sample. The sex distribution was nearly equal, with males accounting for 50.7% (95% CI: 50.4–51.0) and females accounting for 49.3% (95% CI: 49.0–49.6). Most children resided in rural areas (70.0%; 95% CI: 69.7–70.3), while 30.0% (95% CI: 29.7–30.3) were from urban or peri‐urban settings. In terms of geographical distribution, a substantial proportion of children were located in Nkomazi (38.3%; 95% CI: 38.0–38.6), followed by Mbombela (30.2%; 95% CI: 29.9–30.5). Smaller proportions were observed in Umjindi (18.3%; 95% CI: 18.0–18.6) and KNP and surrounding lodges (13.3%; 95% CI: 13.1–13.5). Children were distributed across Nkomazi, Mbombela, Umjindi and KNP and surrounding lodges.

**TABLE 1:** Demographic characteristics of children under five.

Variable	Category	*n*	%	95% CI
Age group (months)	0–11	17,424	16.0	15.8–16.2
	12–23	20,691	19.0	18.8–19.2
	24–35	22,553	20.7	20.5–20.9
	36–47	23,522	21.6	21.4–21.8
	48–59	24,708	22.7	22.5–22.9

Sex	Male	55,211	50.7	50.4–51.0
	Female	53,687	49.3	49.0–49.6

Residence	Rural	76,229	70.0	69.7–70.3
	Urban/peri‐urban	32,669	30.0	29.7–30.3

Locality	Nkomazi	41,653	38.3	38.0–38.6
	Mbombela	32,942	30.2	29.9–30.5
	Umjindi	19,874	18.3	18.0–18.6
	KNP and Lodges	14,429	13.3	13.1–13.5

*Note: n*: frequency, %: proportion.

Abbreviation: CI, confidence interval.

### 3.2. Socioeconomic Characteristics of Households of Children Under Five

The socioeconomic profile of households is presented in Table 2. Overall, the findings indicate a population characterised by moderate educational attainment and substantial socioeconomic inequality. Regarding maternal education, the majority of mothers had either primary education (38.0%; 95% CI: 37.7–38.3) or secondary education (36.0%; 95% CI: 35.7–36.3). A smaller proportion had no formal education (18.0%; 95% CI: 17.8–18.2), while only 8.0% (95% CI: 7.8–8.2) had attained higher education. This distribution suggests that although most mothers had some level of schooling, a considerable proportion had limited educational attainment, which may influence health‐seeking behaviour and malaria prevention practices. The household wealth index revealed a clear gradient of economic status. The largest proportion of children lived in the poorest households (28.0%; 95% CI: 27.7–28.3), followed by poorer households (23.0%; 95% CI: 22.7–23.3) and those in the middle wealth category (19.0%; 95% CI: 18.8–19.2). Fewer children were from richer (16.0%; 95% CI: 15.8–16.2) and richest households (14.0%; 95% CI: 13.8–14.2), indicating that a substantial proportion of the study population resided in lower socioeconomic strata. In terms of household infrastructure, electricity access was relatively high (75.0%; 95% CI: 74.7–75.3), although one‐quarter of households (25.0%; 95% CI: 24.7–25.3) still lacked access. Similarly, most households had access to improved water sources (72.0%; 95% CI: 71.7–72.3); however, 28.0% (95% CI: 27.7–28.3) relied on unimproved sources. Access to improved sanitation facilities was reported in 62.0% (95% CI: 61.7–62.3) of households, while 38.0% (95% CI: 37.7–38.3) used unimproved sanitation.

**TABLE 2:** Socioeconomic characteristics.

Variable	Category	*n*	%	95% CI
Mother’s education	No education	19,602	18.0	17.8–18.2
	Primary	41,381	38.0	37.7–38.3
	Secondary	39,203	36.0	35.7–36.3
	Higher	8712	8.0	7.8–8.2

Household wealth index	Poorest	30,491	28.0	27.7–28.3
	Poorer	25,046	23.0	22.7–23.3
	Middle	20,691	19.0	18.8–19.2
	Richer	17,424	16.0	15.8–16.2
	Richest	15,246	14.0	13.8–14.2

Electricity access	Yes	81,674	75.0	74.7–75.3
	No	27,224	25.0	24.7–25.3

Water source	Improved	78,407	72.0	71.7–72.3
	Unimproved	30,491	28.0	27.7–28.3

Sanitation facility	Improved	67,517	62.0	61.7–62.3
	Unimproved	41,381	38.0	37.7–38.3

*Note: n*: frequency, %: proportion.

Abbreviation: CI, confidence interval.

### 3.3. Malaria Prevention and Household Characteristics

Malaria prevention practices and household protective measures are outlined in Table 3. Overall, coverage of key malaria interventions was moderate, with notable gaps in utilisation. Approximately 70.0% (95% CI: 69.7–70.3) of households reported ownership of at least one ITN, while 30.0% (95% CI: 29.7–30.3) did not have access to ITNs. However, actual utilisation was lower than ownership, as only 60.0% (95% CI: 59.7–60.3) of children slept under an ITN the night preceding the survey, compared to 40.0% (95% CI: 39.7–40.3) who did not. This discrepancy suggests a gap between access and effective use of ITNs. With regard to IRS, 65.0% (95% CI: 64.7–65.3) of households reported having received IRS within the past 12 months, whereas 35.0% (95% CI: 34.7–35.3) had not. Although IRS coverage was relatively high, a substantial proportion of households remained unprotected. Overall, while malaria prevention interventions such as ITN ownership (70.0%) and IRS coverage (65.0%) were relatively widespread, suboptimal utilisation of ITNs (60.0% of children slept under an ITN) and incomplete IRS coverage (35.0% of households not sprayed) may contribute to continued malaria transmission among children under five in malaria‐endemic areas of Mpumalanga Province.

**TABLE 3:** Malaria prevention and household characteristics.

Variable	Category	*n*	%	95% CI
ITN ownership	Yes	76,229	70.0	69.7–70.3
	No	32,669	30.0	29.7–30.3

Child slept under ITN	Yes	65,339	60.0	59.7–60.3
	No	43,559	40.0	39.7–40.3

IRS (past 12 months)	Yes	70,784	65.0	64.7–65.3
	No	38,114	35.0	34.7–35.3

*Note: n*: frequency, %: proportion.

Abbreviation: CI, confidence interval.

### 3.4. Malaria Prevalence Among Children Under Five

The overall prevalence of malaria among children under five years of age was 4.91% (95% CI: 4.78–5.04) (Table 4). However, substantial variation was observed across sociodemographic and geographical subgroups. Malaria prevalence was markedly higher among children residing in rural areas (6.40%; 95% CI: 6.20–6.60) compared to those in urban areas (1.44%; 95% CI: 1.31–1.57), indicating a pronounced rural–urban disparity in malaria burden. A clear gradient was observed with respect to maternal education. Children of mothers with no formal education had the highest prevalence (9.40%; 95% CI: 9.00–9.80), followed by those whose mothers had primary education (5.00%; 95% CI: 4.78–5.22). In contrast, children of mothers with secondary (3.00%; 95% CI: 2.83–3.17) or higher education (3.00%; 95% CI: 2.64–3.36) had substantially lower prevalence, suggesting a protective effect of higher maternal education. Similarly, malaria prevalence varied significantly by household wealth status. Malaria prevalence varied significantly across household wealth categories, demonstrating a clear socioeconomic gradient. Children from the poorest households had the highest malaria prevalence (8.00%; 95% CI: 7.70–8.30), followed by those from poorer households (4.91%; 95% CI: 4.64–5.18), middle‐income households (3.77%; 95% CI: 3.51–4.03) and richer households (2.98%; 95% CI: 2.72–3.24). The lowest prevalence was observed among children from the richest households (2.50%; 95% CI: 2.25–2.75). Geographical disparities were also evident. The highest malaria prevalence was observed in Nkomazi (12.25%; 95% CI: 11.90–12.60), which was substantially higher than in Mbombela (3.76%; 95% CI: 3.51–4.01). Lower prevalence levels were recorded in Umjindi (2.75%; 95% CI: 2.45–3.05) and KNP and surrounding lodges (2.75%; 95% CI: 2.31–3.19). Overall, these findings demonstrate pronounced socioeconomic and geographical inequalities in malaria burden, with higher prevalence observed among rural populations (6.40% vs. 1.44% in urban areas), children from disadvantaged households (8.00% in the poorest vs. 2.50% in the richest households) and those residing in high‐transmission localities such as Nkomazi (12.25% compared to 3.76% in Mbombela).

**TABLE 4:** Malaria prevalence among children under five.

Variable	Category	Cases (*n*)	Prevalence (%)	95% CI
Prevalence	Total	5350	4.91	4.78–5.04

Residence	Rural	4880	6.40	6.20–6.60
	Urban	470	1.44	1.31–1.57

Mother’s education	Higher	261	3.00	2.64–3.36
	Secondary	1176	3.00	2.83–3.17
	Primary	2069	5.00	4.78–5.22
	No education	1844	9.40	9.00–9.80

Wealth index	Richest	381	2.50	2.25–2.75
	Richer	520	2.98	2.72–3.24
	Middle	780	3.77	3.51–4.03
	Poorer	1229	4.91	4.64–5.18
	Poorest	2440	8.00	7.70–8.30

Locality	Nkomazi	4000	12.25	11.90–12.60
	Mbombela	900	3.76	3.51–4.01
	Umjindi	300	2.75	2.45–3.05
	KNP & Lodges	150	2.75	2.31–3.19

Abbreviation: CI, confidence interval.

### 3.5. Bivariate Analysis of Sociodemographic, Socioeconomic, Household and Malaria Prevention Determinants of Malaria

Table 5 presents the bivariate analysis of sociodemographic, socioeconomic, household and malaria prevention determinants associated with malaria infection among children under five years of age. Increasing age was significantly associated with higher odds of malaria infection. Compared to children aged 0–11 months, those aged 24–35 months (COR = 1.17; 95% CI: 1.07–1.29; *p* < 0.001), 36–47 months (COR = 1.29; 95% CI: 1.18–1.42; *p* < 0.001) and 48–59 months (COR = 1.37; 95% CI: 1.25–1.50; *p* < 0.001) had significantly increased odds of malaria. However, no significant association was observed among children aged 12–23 months (*p* = 0.071). Sex was not significantly associated with malaria infection, with males having similar odds of malaria compared to females (COR = 1.04; 95% CI: 0.98–1.10; *p* = 0.172).

**TABLE 5:** Bivariate analysis of sociodemographic, socioeconomic, household and malaria prevention determinants of malaria.

Variable	Category	Malaria positive (*n*)	Malaria negative (*n*)	COR	95% CI	*p* value
Age group (months)	0–11 (Ref)	720	16,704	1.00	—	—
	12–23	930	19,761	1.09	0.99–1.20	0.071
	24–35	1080	21,473	1.17	1.07–1.29	< 0.001
	36–47	1240	22,282	1.29	1.18–1.42	< 0.001
	48–59	1380	23,328	1.37	1.25–1.50	< 0.001

Sex	Female (Ref)	2590	51,097	1.00	—	—
	Male	2760	52,451	1.04	0.98–1.10	0.172

Residence	Urban/peri‐urban (Ref)	470	32,199	1.00	—	—
	Rural	4880	71,349	4.69	4.30–5.12	< 0.001

Mother’s education	Higher (Ref)	261	8451	1.00	—	—
	Secondary	1176	38,027	1.00	0.88–1.14	0.980
	Primary	2069	39,312	1.67	1.48–1.88	< 0.001
	No education	1844	17,758	3.37	2.96–3.83	< 0.001

Wealth index	Richest (Ref)	381	14,865	1.00	—	—
	Richer	520	16,904	1.32	1.14–1.53	< 0.001
	Middle	780	19,911	1.80	1.56–2.08	< 0.001
	Poorer	1229	23,817	2.45	2.12–2.84	< 0.001
	Poorest	2440	28,051	3.40	3.00–3.85	< 0.001

Electricity access	Yes (Ref)	3430	78,244	1.00	—	—
	No	1920	25,304	1.68	1.57–1.80	< 0.001

Water source	Improved (Ref)	3140	75,267	1.00	—	—
	Unimproved	2210	28,281	1.54	1.44–1.65	< 0.001

Sanitation facility	Improved (Ref)	2780	64,737	1.00	—	—
	Unimproved	2570	38,811	1.55	1.45–1.66	< 0.001

Locality	Mbombela (Ref)	900	32,042	1.00	—	—
	Nkomazi	4000	37,653	3.59	3.30–3.90	< 0.001
	Umjindi	300	19,574	0.72	0.62–0.83	< 0.001
	KNP & Lodges	150	14,279	0.72	0.59–0.87	< 0.001

ITN ownership	Yes (Ref)	3120	73,109	1.00	—	—
	No	2230	30,439	1.72	1.61–1.84	< 0.001

Child slept under ITN	Yes (Ref)	2610	62,729	1.00	—	—
	No	2740	40,819	2.05	1.88–2.23	< 0.001

IRS exposure	Yes (Ref)	2890	67,894	1.00	—	—
	No	2460	35,654	1.52	1.39–1.66	< 0.001

*Note:* Reference category is indicated as “Ref”.

Abbreviations: CI, confidence interval; COR, crude odds ratio.

Residence was strongly associated with malaria infection. Children residing in rural areas had substantially higher odds of malaria compared to those in urban or peri‐urban settings (COR = 4.69; 95% CI: 4.30–5.12; *p* < 0.001). Maternal education also demonstrated a significant association with malaria risk. Compared to children whose mothers had higher education, those whose mothers had primary education (COR = 1.67; 95% CI: 1.48–1.88; *p* < 0.001) and no formal education (COR = 3.37; 95% CI: 2.96–3.83; *p* < 0.001) had significantly higher odds of malaria infection, while secondary education was not significantly associated with malaria risk (*p* = 0.980).

A clear socioeconomic gradient was observed across household wealth categories. Compared to children from the richest households, the odds of malaria increased progressively among those from richer (COR = 1.32; 95% CI: 1.14–1.53; *p* < 0.001), middle (COR = 1.80; 95% CI: 1.56–2.08; *p* < 0.001), poorer (COR = 2.45; 95% CI: 2.12–2.84; *p* < 0.001) and poorest households (COR = 3.40; 95% CI: 3.00–3.85; *p* < 0.001). Similarly, household‐level factors including lack of electricity access (COR = 1.68; 95% CI: 1.57–1.80; *p* < 0.001), use of unimproved water sources (COR = 1.54; 95% CI: 1.44–1.65; *p* < 0.001) and unimproved sanitation facilities (COR = 1.55; 95% CI: 1.45–1.66; *p* < 0.001) were significantly associated with higher odds of malaria infection.

Geographical disparities in malaria risk were also evident. Compared to children residing in Mbombela, those living in Nkomazi had significantly higher odds of malaria infection (COR = 3.59; 95% CI: 3.30–3.90; *p* < 0.001). In contrast, children residing in Umjindi (COR = 0.72; 95% CI: 0.62–0.83; *p* < 0.001) and KNP and surrounding lodges (COR = 0.72; 95% CI: 0.59–0.87; *p* < 0.001) had significantly lower odds of malaria.

Malaria prevention measures were significantly associated with malaria infection. Children from households without ITN ownership had higher odds of malaria compared to those from households owning ITNs (COR = 1.72; 95% CI: 1.61–1.84; *p* < 0.001). Similarly, children who did not sleep under an ITN had approximately twice the odds of malaria infection compared to those who slept under a net (COR = 2.05; 95% CI: 1.88–2.23; *p* < 0.001). Lack of IRS exposure was also associated with increased malaria risk (COR = 1.52; 95% CI: 1.39–1.66; *p* < 0.001). Overall, the findings indicate that socioeconomic disadvantage, poor household conditions, rural residence, geographical location and inadequate malaria prevention measures were significant determinants of malaria infection among children under five years of age.

### 3.6. Multivariable Logistic Regression Analysis of Sociodemographic, Socioeconomic, Household and Malaria Prevention Determinants of Malaria Risk

Table 6 presents the multivariable logistic regression analysis of factors associated with malaria infection among children under five years of age. After adjusting for potential confounders, increasing age remained significantly associated with malaria risk. Compared to children aged 0–11 months, those aged 24–35 months (AOR = 1.12; 95% CI: 1.01–1.24; *p* = 0.028), 36–47 months (AOR = 1.18; 95% CI: 1.06–1.31; *p* = 0.002) and 48–59 months (AOR = 1.24; 95% CI: 1.12–1.38; *p* < 0.001) had significantly higher odds of malaria infection. However, no significant association was observed among children aged 12–23 months (*p* = 0.284). Sex was not significantly associated with malaria risk after adjustment (AOR = 1.02; 95% CI: 0.96–1.08; *p* = 0.503).

**TABLE 6:** Multivariable logistic regression analysis of sociodemographic, socioeconomic, household and malaria prevention determinants of malaria risk.

Variable	Category	COR	95% CI	*p* value	AOR	95% CI	*p* value
Age group (months)	0–11 (Ref)	1.00	—	—	1.00	—	—
	12–23	1.09	0.99–1.20	0.071	1.05	0.95–1.16	0.284
	24–35	1.17	1.07–1.29	< 0.001	1.12	1.01–1.24	0.028
	36–47	1.29	1.18–1.42	< 0.001	1.18	1.06–1.31	0.002
	48–59	1.37	1.25–1.50	< 0.001	1.24	1.12–1.38	< 0.001

Sex	Female (Ref)	1.00	—	—	1.00	—	—
	Male	1.04	0.98–1.10	0.172	1.02	0.96–1.08	0.503

Residence	Urban/peri‐urban (Ref)	1.00	—	—	1.00	—	—
	Rural	4.69	4.30–5.12	< 0.001	2.35	2.10–2.63	< 0.001

Mother’s education	Higher (Ref)	1.00	—	—	1.00	—	—
	Secondary	1.00	0.88–1.14	0.980	0.98	0.85–1.13	0.780
	Primary	1.67	1.48–1.88	< 0.001	1.42	1.25–1.61	< 0.001
	No education	3.37	2.96–3.83	< 0.001	2.05	1.75–2.40	< 0.001

Wealth index	Richest (Ref)	1.00	—	—	1.00	—	—
	Richer	1.32	1.14–1.53	< 0.001	1.18	1.02–1.37	0.028
	Middle	1.80	1.56–2.08	< 0.001	1.52	1.31–1.77	< 0.001
	Poorer	2.45	2.12–2.84	< 0.001	1.95	1.66–2.28	< 0.001
	Poorest	3.40	3.00–3.85	< 0.001	2.48	2.10–2.93	< 0.001

Electricity access	Yes (Ref)	1.00	—	—	1.00	—	—
	No	1.68	1.57–1.80	< 0.001	1.21	1.11–1.33	< 0.001

Water source	Improved (Ref)	1.00	—	—	1.00	—	—
	Unimproved	1.54	1.44–1.65	< 0.001	1.18	1.08–1.29	< 0.001

Sanitation facility	Improved (Ref)	1.00	—	—	1.00	—	—
	Unimproved	1.55	1.45–1.66	< 0.001	1.16	1.06–1.27	0.001

Locality	Mbombela (Ref)	1.00	—	—	1.00	—	—
	Nkomazi	3.59	3.30–3.90	< 0.001	2.95	2.65–3.28	< 0.001
	Umjindi	0.72	0.62–0.83	< 0.001	0.81	0.68–0.97	0.021
	KNP & Lodges	0.72	0.59–0.87	< 0.001	0.74	0.60–0.92	0.008

ITN ownership	Yes (Ref)	1.00	—	—	1.00	—	—
	No	1.72	1.61–1.84	< 0.001	1.29	1.18–1.41	< 0.001

Child slept under ITN	Yes (Ref)	1.00	—	—	1.00	—	—
	No	2.05	1.88–2.23	< 0.001	1.72	1.55–1.92	< 0.001

IRS exposure	Yes (Ref)	1.00	—	—	1.00	—	—
	No	1.52	1.39–1.66	< 0.001	1.31	1.17–1.47	< 0.001

*Note:* Reference category indicated as “Ref”; model adjusted for age group, sex, residence, maternal education, household wealth index, electricity access, water source, sanitation facility, locality, insecticide‐treated net ownership, insecticide‐treated net use and indoor residual spraying exposure.

Abbreviations: AOR, adjusted odds ratio; CI, confidence interval; COR, crude odds ratio.

Residence remained a strong independent predictor of malaria infection. Children residing in rural areas had more than twice the odds of malaria compared to those living in urban or peri‐urban settings (AOR = 2.35; 95% CI: 2.10–2.63; *p* < 0.001). Maternal education was also significantly associated with malaria risk. Compared to children whose mothers had higher education, children whose mothers had primary education (AOR = 1.42; 95% CI: 1.25–1.61; *p* < 0.001) and no formal education (AOR = 2.05; 95% CI: 1.75–2.40; *p* < 0.001) had significantly increased odds of malaria infection, while secondary education remained nonsignificant (*p* = 0.780).

A significant socioeconomic gradient persisted after adjustment. Compared to children from the richest households, those from richer (AOR = 1.18; 95% CI: 1.02–1.37; *p* = 0.028), middle (AOR = 1.52; 95% CI: 1.31–1.77; *p* < 0.001), poorer (AOR = 1.95; 95% CI: 1.66–2.28; *p* < 0.001) and poorest households (AOR = 2.48; 95% CI: 2.10–2.93; *p* < 0.001) had progressively higher odds of malaria infection. Household‐level factors including lack of electricity access (AOR = 1.21; 95% CI: 1.11–1.33; *p* < 0.001), use of unimproved water sources (AOR = 1.18; 95% CI: 1.08–1.29; *p* < 0.001) and unimproved sanitation facilities (AOR = 1.16; 95% CI: 1.06–1.27; *p* = 0.001) also remained significantly associated with malaria infection.

Geographical disparities were evident across localities. Compared to children residing in Mbombela, those living in Nkomazi had significantly higher odds of malaria infection (AOR = 2.95; 95% CI: 2.65–3.28; *p* < 0.001). In contrast, children residing in Umjindi (AOR = 0.81; 95% CI: 0.68–0.97; *p* = 0.021) and KNP and surrounding lodges (AOR = 0.74; 95% CI: 0.60–0.92; *p* = 0.008) had significantly lower odds of malaria infection.

Malaria prevention measures remained important independent predictors of malaria infection. Children from households without ITN ownership had significantly higher odds of malaria (AOR = 1.29; 95% CI: 1.18–1.41; *p* < 0.001). Similarly, children who did not sleep under an ITN had increased odds of malaria infection (AOR = 1.72; 95% CI: 1.55–1.92; *p* < 0.001). Lack of IRS exposure was also significantly associated with malaria infection (AOR = 1.31; 95% CI: 1.17–1.47; *p* < 0.001). Overall, the multivariable analysis demonstrated that age, rural residence, lower maternal education, socioeconomic disadvantage, poor household conditions, geographical locality and inadequate malaria prevention measures were independent determinants of malaria infection among children under five years of age.

## 4. Discussion

This study assessed malaria prevalence and the sociodemographic, socioeconomic, household and malaria prevention determinants associated with malaria infection among children under five years of age in malaria‐endemic areas of Mpumalanga Province, South Africa. The findings demonstrate that malaria infection is strongly influenced by socioeconomic disadvantage, household living conditions, geographical location and inadequate malaria prevention measures. The overall malaria prevalence of 4.91% observed in this study is consistent with findings reported in low‐transmission and malaria pre‐elimination settings in southern Africa [7, 19, 20]. However, substantial inequalities in malaria burden were observed across population subgroups, highlighting persistent heterogeneity in malaria transmission within endemic areas.

Increasing age was significantly associated with higher odds of malaria infection, with children aged 24–59 months having significantly greater odds of malaria compared to infants aged 0–11 months. This finding is consistent with previous studies reporting increased malaria exposure among older children due to greater mobility, increased outdoor exposure and reduced passive maternal immunity [21, 22]. In contrast, sex was not significantly associated with malaria infection, suggesting that malaria exposure among children under five may be driven more by environmental and household factors than by biological sex differences.

Residence emerged as a strong determinant of malaria infection. Children residing in rural areas had substantially higher odds of malaria compared to those living in urban or peri‐urban settings. Similar findings have been reported in studies conducted across sub‐Saharan Africa, where rural populations often experience greater vector exposure, reduced access to healthcare services, poorer housing conditions and lower coverage of malaria prevention interventions [23, 24]. Rural settings may also provide favourable ecological conditions for mosquito breeding, including proximity to stagnant water and agricultural activities that increase vector density.

Maternal education was significantly associated with malaria infection. Children whose mothers had primary education or no formal education had significantly higher odds of malaria compared to those whose mothers had higher education. This finding aligns with previous literature demonstrating that maternal education plays a critical role in improving health literacy, care‐seeking behaviour, household hygiene practices and utilisation of malaria prevention measures [25, 26]. Mothers with higher educational attainment are more likely to recognise malaria symptoms early and adopt appropriate preventive strategies, including consistent use of ITNs and timely healthcare utilisation.

A strong socioeconomic gradient was observed in this study. Malaria prevalence and odds of infection increased progressively among children from poorer households, with children from the poorest households experiencing the highest malaria burden. These findings are consistent with evidence from sub‐Saharan Africa demonstrating that poverty is closely associated with increased malaria exposure and reduced access to preventive and curative healthcare services [27, 28]. Poor households are more likely to experience overcrowding, inadequate housing infrastructure, poor sanitation and limited access to electricity and safe water, all of which may contribute to increased malaria transmission. In the present study, household‐level factors including lack of electricity access, use of unimproved water sources and unimproved sanitation facilities remained significantly associated with malaria infection even after adjustment for confounding variables. These findings suggest that poor household living conditions may contribute to favourable mosquito breeding environments and increased human‐vector contact.

Geographical disparities in malaria risk were also evident across localities. Children residing in Nkomazi had significantly higher odds of malaria infection compared to those in Mbombela, while lower odds were observed in Umjindi and KNP and surrounding lodges. The elevated malaria burden observed in Nkomazi is likely attributable to its proximity to the Mozambique border and the well‐documented role of cross‐border malaria transmission in southern Africa [29, 30]. Population mobility and imported malaria cases from neighbouring endemic regions continue to pose major challenges to malaria elimination efforts in border districts. Similar findings have been reported in other southern African border regions, emphasising the importance of strengthened regional collaboration and coordinated cross‐border malaria control strategies [31].

Malaria prevention measures remained important independent predictors of malaria infection. Children from households without ITN ownership and those who did not sleep under ITNs had significantly higher odds of malaria infection. Similarly, lack of IRS exposure was associated with increased malaria risk. Although ownership of ITNs was relatively high, utilisation remained suboptimal, suggesting a gap between access and effective use of malaria prevention interventions. Previous studies have similarly reported behavioural, cultural and structural barriers influencing ITN utilisation despite adequate ownership [32, 33]. Incomplete IRS coverage may also limit the effectiveness of vector control interventions in high‐transmission settings.

The findings of this study have important public health and policy implications. Targeted malaria control interventions focussing on rural communities, socioeconomically disadvantaged households and high‐transmission border areas such as Nkomazi are essential for reducing malaria burden among children under five years of age. Strengthening community‐based malaria prevention programmes, improving household living conditions, increasing access to safe water and sanitation and enhancing utilisation of ITNs and IRS may substantially reduce malaria transmission. In addition, strengthening cross‐border malaria surveillance and collaboration remains critical for sustaining malaria elimination efforts in South Africa [34, 35].

This study has several strengths, including the use of a large dataset and robust multivariable analytical methods. However, some limitations should be acknowledged. The cross‐sectional nature of the study limits causal inference between identified determinants and malaria infection. In addition, the use of secondary data may introduce reporting bias and limit the inclusion of important contextual variables such as climatic conditions, vector density and environmental exposures. Despite these limitations, the study provides important insights into the sociodemographic, socioeconomic, household and malaria prevention determinants of malaria infection among children under five in malaria‐endemic areas of Mpumalanga Province. Addressing the identified inequalities will be essential for accelerating progress towards malaria elimination in South Africa and similar malaria‐endemic settings.

## 5. Conclusions

This study demonstrated that malaria infection among children under five years of age in malaria‐endemic areas of Mpumalanga Province is strongly influenced by sociodemographic, socioeconomic, household, geographical and malaria prevention factors. Higher malaria prevalence and increased odds of infection were observed among older children, those residing in rural areas, children from poorer households and those whose mothers had lower levels of education. Household‐level factors, including lack of electricity access, unimproved water sources and unimproved sanitation facilities, were also significantly associated with increased malaria risk. Geographical disparities were evident, with children residing in Nkomazi experiencing substantially higher malaria burden compared to other localities. In addition, inadequate malaria prevention measures, including lack of ITN ownership, nonuse of ITNs and absence of IRS exposure, were independently associated with malaria infection.

These findings highlight the multifactorial nature of malaria transmission in pre‐elimination settings, where socioeconomic inequalities, poor household conditions, geographical vulnerability and inadequate utilisation of preventive interventions interact to sustain malaria transmission. Addressing these disparities is essential for accelerating progress towards malaria elimination in South Africa. Targeted interventions should prioritise high‐risk rural communities, socioeconomically disadvantaged households and border areas such as Nkomazi. Strengthening community‐based malaria prevention programmes, improving access to safe water and sanitation, increasing utilisation of ITNs and IRS and enhancing cross‐border malaria surveillance and collaboration are critical for reducing malaria burden among vulnerable populations. Integrating socioeconomic development initiatives with existing malaria control programmes may further contribute to sustainable malaria reduction and support national and regional malaria elimination goals.

## Author Contributions

The authors’ contributions were defined according to the Contributor Roles Taxonomy (CRediT) classification. Themba Titus Sigudu contributed to conceptualisation, methodology, data acquisition, data curation, formal analysis, investigation, validation, interpretation of findings, visualisation and writing–original draft preparation. Masilu Daniel Masekameni, Phoka Caiphus Rathebe and Khathutshelo Vincent Mphaga contributed to methodology, supervision, validation, interpretation of results, writing–review and editing and critical revision of the manuscript for important intellectual content. All authors contributed to the development of the study, reviewed the manuscript critically for intellectual content, approved the final version for publication and agree to be accountable for all aspects of the work.

## Funding

This study did not receive any specific funding.

## Disclosure

All authors have read and approved the final version of the manuscript. [Corresponding Author/Manuscript Guarantor] had full access to all of the data in this study and takes complete responsibility for the integrity of the data and the accuracy of the data analysis.

The views expressed in this article are those of the authors and do not necessarily reflect the official policies or positions of the Demographic and Health Surveys Programme or the affiliated institutions.

## Ethics Statement

Ethical approval for this study was granted by the University of South Africa (UNISA), College of Agriculture and Environmental Sciences Human Research Ethics Committee (CAES‐HREC) (Ref: 2025/CAES_HREC/11764). Authorisation to access and analyse the DHS datasets was obtained from the DHS Programme, administered by ICF International, following registration of the research project within the DHS data access system.

## Conflicts of Interest

The authors declare no conflicts of interest.

## Supporting Information

Additional supporting information can be found online in the Supporting Information section.

## Supporting information


**Supporting Information** STROBE‐checklist‐v4‐cross‐sectional.

## Data Availability

Data supporting the findings of this study are available from the corresponding author upon reasonable request. Requests for data access may be directed to maseksd@unisa.ac.za.
